# Screening of High Temperature-Tolerant Oleaginous Diatoms

**DOI:** 10.4014/jmb.2002.02053

**Published:** 2020-04-23

**Authors:** Lingxiang Zhang, Fan Hu, Xiu Wan, Yufang Pan, Hanhua Hu

**Affiliations:** 1 Key Laboratory of Algal Biology, Institute of Hydrobiology, Chinese Academy of Sciences, Wuhan 430072, P.R. China; 2 School of Foreign Languages, China University of Geosciences, Wuhan 430074, P.R. China; 3 University of Chinese Academy of Sciences, Beijing 100049, P.R. China

**Keywords:** Diatom, high temperature, triacylglycerol, fatty acids, lipid production

## Abstract

Screening suitable strains with high temperature adaptability is of great importance for reducing the cost of temperature control in microalgae cultivation, especially in summer. To obtain high temperature-tolerant diatoms, water samples were collected in summer from 7 different regions of China across the Northeast, North and East. A total of 731 water samples was collected and from them 131 diatom strains were isolated and identified based on the 18S rRNA sequences. Forty-nine strains out of the 131 diatoms could survive at 30°C, and 6 strains with relatively high biomass and lipid content at high temperature were selected and were found to be able to grow at 35°C. *Cyclotella* sp. HB162 had the highest dry biomass of 0.46 g/l and relatively high triacylglycerol (TAG) content of 237.4 mg/g dry biomass. The highest TAG content of 246.4 mg/g dry biomass was obtained in *Fistulifera* sp. HB236, while *Nitzschia palea* HB170 had high dry biomass (0.33 g/l) but relatively low TAG content (105.9 mg/g dry biomass). *N. palea* HB170 and *Fistulifera* sp. HB236 presented relatively stable growth rates and lipid yields under fluctuating temperatures ranging from 28 to 35°C, while *Cyclotella* HB162 maintained high lipid yield at temperatures below 25°C. The percentage of saturated fatty acids and monounsaturated fatty acids in all the 6 strains was 84-91% in total lipids and 90-94% in TAGs, which makes them the ideal feedstock for biodiesel.

## Introduction

With the exhaustion of fossil fuels and the aggravation of environmental problems from fossil fuels usage, renewable and environmentally friendly energy sources are being explored [1]. Biodiesel, referred to as fatty acid methyl esters (FAMEs) and fatty acid ethyl esters, is recognized as an ideal substitute for fossil fuels to solve the energy crisis [2]. Conventional biodiesel is mainly produced from vegetable oils or animal fats [3]. Nevertheless, the large-scale production of biodiesel from them adversely impacted supplies of food and other agricultural products, leading to an increase in the price of biodiesel and impeding its usage [3, 4]. Microalgae, as the most promising feedstock for biodiesel production, have attracted considerable attention [5]. The main attractions of using microalgae for biodiesel production are the short growth cycle, high biomass and lipid content, less occupied land, easier cultivation and ease of being modified by biotechnological means [5-7].

Screening high quality microalgae and determining the microalgae oil production efficiency are the basis and core of microalgae energy development [8]. Until now, many algal species have been selected as potential feedstock for mass biodiesel production, such as industrial oleaginous *Nannochloropsis* spp., *Chlorella* species including *Chlorella protothecoides* and *Chlorella vulgaris*, diatoms *Cyclotella cryptica* and *Phaeodactylum tricornutum*, and so on [9-13]. Diatoms have been regarded as competent strains for biofuel production due to their superiorities, including rapid multiplication, short life spans, and higher productivity ecologically than other algal classes [14]. They are at least on a par with, if not superior to, other classes of algae in terms of their ability to accumulate lipids, and they can rapidly induce triacylglycerol (TAG) accumulation under Si limitation [14]. Diatoms, responsible for about 20% of the primary production on Earth, are not only widely distributed but also highly diverse with an estimation of over 100,000 species [15].

Growth rate, lipid productivity and fatty acid composition of diatoms are influenced tremendously by temperatures, thus temperature adaptation is of great importance for screening strains to ensure high lipid productivity [16]. Diatoms usually thrive in cold seasons and are not suitable for outdoor mass cultivation in hot seasons. With increasing temperature, the proportions of saturated fatty acids (SAFA) and monounsaturated fatty acids (MUFA) have increased significantly in some microalgae [17], and ideal strains should contain high relative percentages of SAFA and MUFA for production of high-quality biodiesel [18, 19]. Therefore, the ability of diatoms to tolerate high temperatures must be taken into account in the screening process, especially in an outdoor mass cultivation [20, 21].

In this study, diatom strains isolated from freshwater in seven provinces of China were screened by thin-layer chromatography (TLC) for a rough identification of high lipid productive strains on the basis of the relative TAG content. High oleaginous diatoms with high temperature tolerance were cultivated at different temperatures. Their growth, total lipid and TAG contents, and fatty acid compositions were evaluated to obtain good diatom strains with inherent tolerance to high or fluctuating temperatures. Three diatom strains, *Cyclotella* sp. HB162, *Fistulifera* sp. HB236 and *Nitzschia palea* HB170, showed good adaptation to high or fluctuating temperatures and could serve as potential feedstock for outdoor biodiesel production.

## Materials and Methods

### Sampling and Isolation

Water samples were collected in summer (mainly from June to September) from reservoirs, rivers, lakes and ponds in the provinces of Liaoning (LN), Jilin (JL), Zhejiang (ZJ), Heilongjiang (HLJ), Hebei (HB), Shanxi (SX) and the Inner Mongolia Autonomous Region (NM) of China. The location, pH, temperature and electrical conductivity of the water bodies were recorded (supplementary data). Algal colonies were isolated from the water samples with CSi medium [22] agar (1.2%) using the spread plate method as described before [21]. Individual colonies were then picked and further purified by repeated streaking on a new plate. Subsequently, these purified unialgal forms were maintained by frequent sub-culturing in CSi medium at 22°C under 30 μmol photons/(m^2^ s) continuous illumination before use.

### Identification of Diatoms

All isolated microalgae strains were initially examined by microscopy and those diatom strains were then identified by 18S rRNA sequence analysis. Total genomic DNA of the diatom strains was extracted according to the manufacturer’s instructions for a glass milk DNA isolation kit (Fermentas, Lithuania). The 18S rRNA gene amplification was carried out, using the general primers 18S-1 (5’-tggttgatcctgccagtagtc-3’) and 18S-2 (5’- tgatccttctgcaggttcacc-3’), by polymerase chain reaction (PCR) as previously described [23]. Agarose gel purification of PCR product was carried out using the Aid Quick Gel Extraction Kit (Aidlab Biotech, China). Then, purified product was cloned into pMD18-T (Takara, China) and sequenced. Sequencing results were aligned using the NCBI Nucleotide BLAST. Phylogenetic analyses were performed by PAUP4.0b [24], with 1000 bootstrap replicates for neighbor-joining and parsimony analyses and with 100 replicates for the maximum- likelihood analysis using *Ulothrix zonata* (Z47999) and *Gloeotilopsis planctonica* (Z28970) as the outgroup.

### Preliminary Screening of High Oleaginous Diatoms

All diatom strains were grown in 50 ml flasks containing 20 ml CSi liquid medium (the starting density is 2 × 10^5^ cells/ml) at 22°C under continuous illumination of 50 μmol photons/(m^2^ s) for 15 d, then total lipids were extracted from the cell pellets and analyzed using TLC for the comparison of the TAG content. TLC was performed as described by Reiser and Somerville [25] on silica gel plates 60 F254 (Merck KgaA, Germany). Triolein (Sigma, USA) was used as the standard for qualitative analysis of TAGs, and the relative quantity of TAGs was evaluated using ImageJ software based on the gray intensity. Total lipids were extracted according to the procedure described by Bligh and Dyer [26] and their contents were determined with gravimetric method. Briefly, the lipids were extracted with chloroform–methanol (1:1, v/v) from 100 mg dry cells and separated into chloroform and aqueous methanol layers by centrifugation. Lipid phase was pipetted to a new tube for evaporation under a gentle stream of nitrogen. The evaporated residue was dried in an oven at 105°C for hours to a constant weight, and then the total lipid contents were determined by the weight method.

### Screening High Temperature Tolerant Diatoms

For preliminary screening of high temperature-tolerant diatom, all strains were grown at 30°C with the starting density of 2× 10^5^ cells/ml for 15 days, and then cell survival was detected by both naked eye and microscope observation. Subsequently, all 49 surviving strains were cultivated in glass tubes (3 cm of inner diameter and 35 cm of height) at 28°C for 10 days. Cell density and size were determined under microscope using a Malassez chamber (0.2 mm). For comparison, relative cell biomass was obtained based on cell density and cell area, and the relative lipid yield was calculated from relative cell biomass and the total lipid content was measured as above.

### Growth of 6 Representative Diatom Strains

Six diatom strains (*Cyclotella* sp. HB162, *Fistulifera* sp. HB236, *Nitzschia palea* HB170, *Nitzschia* sp. ZJ183, *Nitzschia* sp. ZJ184 and *Nitzschia* sp. HLJ48) were selected and cultivated in glass culture tubes containing 180 ml of CSi medium with aeration by sterile air under continuous illumination of 70 μmol photons/(m^2^ s) at different temperatures for 10 days. Cell density was determined by cell counts using the Malassez chamber every 2 days and specific growth rates (μ) were calculated according to μ = (lnX*_t_* − lnX_0_)/t, where X_0_ is the initial cell density and X*_t_* is the cell density after *t* days. Algal dry biomass was determined by the weight method at the end of the experiment.

### Total Lipid and TAG Contents of 6 Representative Diatom Strains

Total lipids of six representative diatom strains from 22, 25, and 28°C cultures were extracted and their contents were determined by weight method. The total lipids from 25°C cultures were resolved by TLC, and TAGs were scraped out and methylated with sulfuric acid-methanol. TAG contents were determined based on all fatty acid methyl ester peak areas using gas chromatography, and methyl heptadecanoic acid (C17:0) was used as an internal standard.

### Fatty Acid Composition of Total Lipid and TAG

Total lipids of 6 representative diatom strains from 25°C cultures were obtained as described above. TAGs were separated by TLC and the band of TAGs on the plates was scraped out and then extracted with chloroform. Total lipids and TAGs were methylated with sulfuric acid-methanol as described before [23]. Gas chromatography of the FAMEs was carried out with a TRACE GC (Thermo Scientific, Italy) equipped with a flame ionization detector, a capillary column (60 m × 0.25 mm) (DB-23, J&W Scientific, USA) and a split/splitless injector. Highly purified N2 gas was used as the carrier gas with a flow rate of 2.0 ml/min. Initial column temperature was set at 50°C and subsequently raised to 170°C at 40°C/min, then raised to 210°C at 18°C/min and held for 28 min. Two microliters of sample were injected into the inlet and FAMEs were identified by chromatographic comparison of their retention time with authentic standards (Sigma). Heptadecanoic acid (C17:0) was used as internal standard. The quantity of individual fatty acids was calculated based on the peak area of a fatty acid species to the total peak area of all the fatty acids in the sample.

## Results and Discussion

### Isolation and Identification of Diatoms

Diatoms are widely distributed in freshwater, and are remarkably dominant species in the cold season during winter and early spring [27]. Therefore, in order to obtain high temperature-tolerant diatoms, water samples were collected in summer from 7 different regions of China across the Northeast, North and East. A total of 731 water samples was collected and from them 131 diatom strains were isolated. Except for 5 unidentified species, 13 strains were from *Achnanthidium*, 1 from *Craticula*, 7 from *Cyclotella*, 23 from *Fistulifera*, 1 from *Gomphonema*, 2 from *Lemnicola*, 65 from *Nitzschia*, 3 from *Pinnularia*, 10 from *Sellaphora* and 1 from *Synedra* based on the morphological characteristics and 18S rRNA gene sequence analysis.

**Fig. 1:**
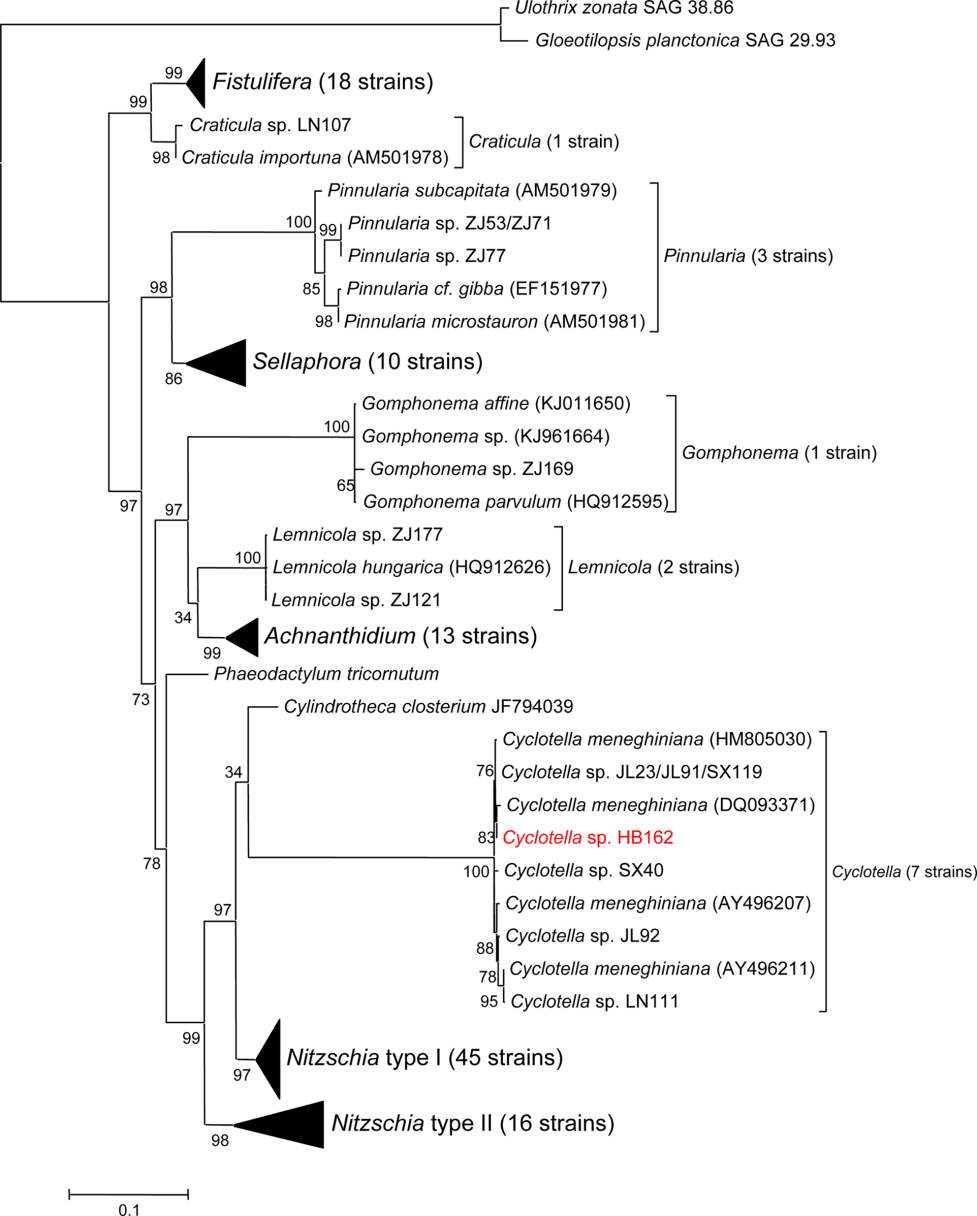
A maximum-likelihood tree based on 18S rRNA gene sequences illustrating the phylogeny of the isolated diatoms. Branch lengths correspond to the evolutionary distances. A distance of 0.1 is indicated by the scale. The selected strain for detailed study is in red.

**Fig. 2:**
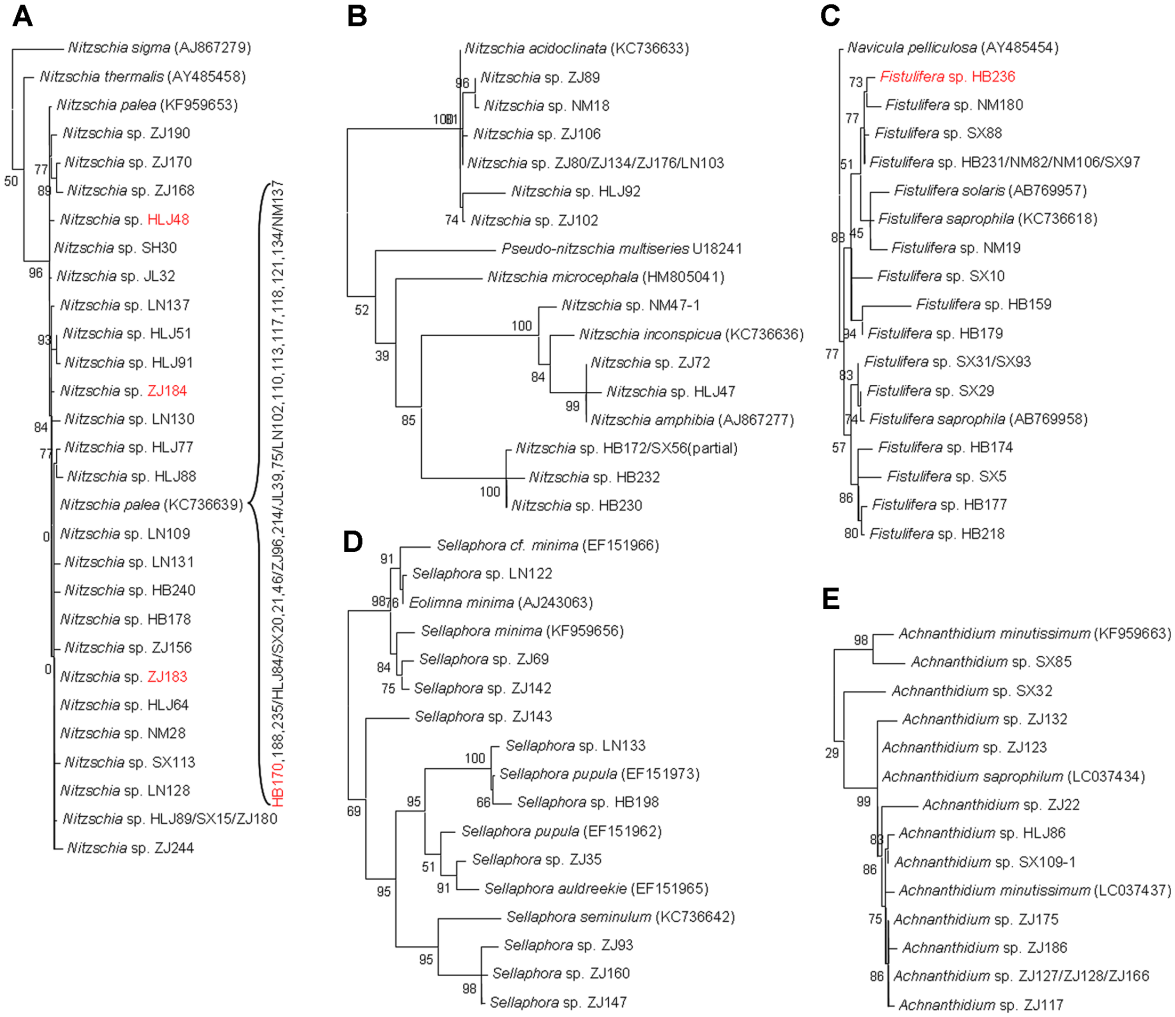
The maximum-likelihood subtree from Fig. 1 showing all isolated strains in genera *Nitzschia* (A and B), *Fistulifera* (C), *Sellaphora* (D), and *Achnanthidium* (E). The selected strains for detailed study are in red.

Phylogenetic trees of our isolated diatoms with known diatom species provided a clear resolution to generic assignment (Fig. 1). Nine genera formed 10 clades. Genus *Nitzschia* was divided into two monophyletic groups, which is consistent with the previous report of Lundholm, *et al*. [28]. One group contained *Nitzschia sigma*, *N. thermalis*, *N. palea* and isolated strains HLJ48, ZJ183, ZJ184, HB170, and etc. (Fig. 2A). The other one contained *Pseudo-Nitzschia multiseries* (Fig. 2B), and *Pseudo-Nitzschia* has been recognized as a genus distinct from *Nitzschia* [29]. Nineteen isolated *Nitzschia* strains, including HB170, HLJ84, SX20, ZJ96, JL39, LN102, and NM137, were found to be identical to *N. palea* in 18S rRNA sequence. The 18S rRNA gene of *Fistulifera* sp. HB236 had 99.3% sequence similarity to that of *Fistulifera solaris* (Fig. 2C), an excellent oleaginous diatom [30]. *Cyclotella* sp. HB162 clustered together with 4 *Cyclotella meneghiniana* strains, and shared an 18S rRNA gene sequence similarity of 99.8% with *C. meneghiniana* HM505030 (Fig. 1).

### Habitats, Total Lipids and TAGs of Isolated Diatoms

**Fig. 3:**
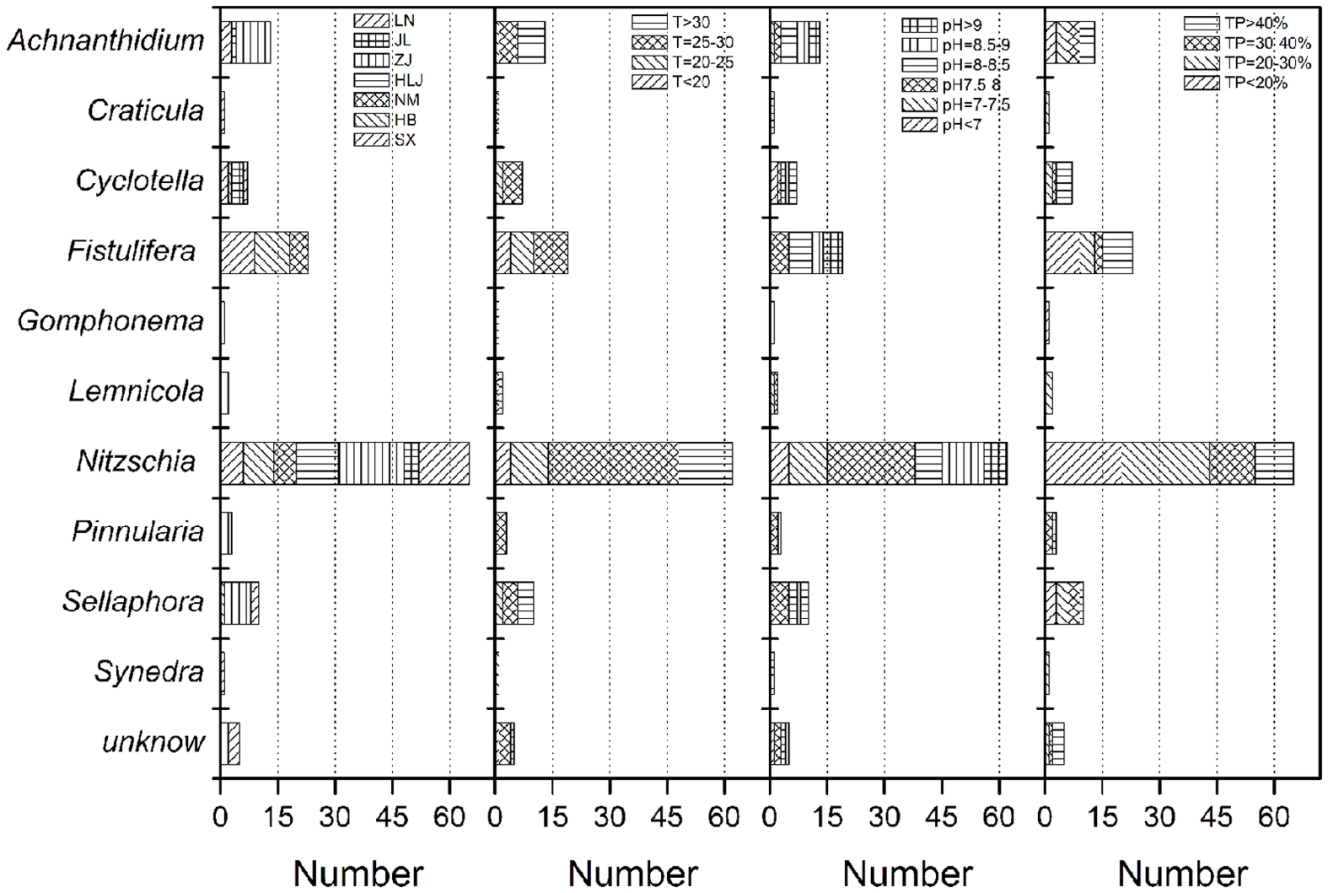
Summary of uni-algal culture of diatoms isolated from different regions in China (LN: Liaoning Province; JL: Jilin Province; ZJ: Zhejiang Province; HLJ: Heilongjiang Province; NM: Inner Mongolia Autonomous region; HB: Hebei Province; SX: Shanxi Province). T and pH represent the water temperature (°C) and pH value of the isolated sites; TP: total lipid content.

Among the 131 diatom strains, 39 were isolated from the Northeast (Liaoning, Jilin, and Heilongjiang Provinces), 51 from the North (Hebei and Shanxi Provinces, and Inner Mongolia Autonomous Region) and 41 from the East (Zhejiang Province). The ecological habitat information on all the strains except 7 diatoms was shown in Fig. 3 and supplementary data, including the geographical location, water temperatures and pH value.

Although 246 and 84 water samples were collected from Zhejiang and Jilin, 41 and 7 diatom strains were obtained from these samples respectively. Only 24 from the former and 19 from the latter were collected at temperatures lower than 25°C with the highest record of 38 and 28°C, respectively. However, 20, 12, and 11 diatom strains were obtained from 58, 50, and 51 water samples collected from Liaoning, Heilongjiang and Inner Mongolia, whose average temperatures were around 20°C. In particular, the lowest temperature was 11°C in Liaoning, 19°C in Inner Mongolia, and 20°C in Heilongjiang. It is indicated that diatoms have a preference for low temperatures and thus thrive in cold waters [27, 31]. In this study, 77% of isolated diatom strains were from waters at temperatures lower than 30°C, while 23% of strains were isolated at above 30°C. It is obvious that many potential high temperature adaptation diatoms can be obtained in summer.

Most of the isolated diatoms (114 strains) prefer alkaline waters, and only 7 strains, including 2 *Cyclotella* strains and 5 *Nitzschia* strains, were isolated from acidic waters. This indicates that most of the diatoms possess efficient CO_2_ concentrating mechanisms (CCMs), and are able to assimilate bicarbonate directly or via extracellular carbonic anhydrases [32]. Strains of *Nitzschia* accounted for a significant proportion (over 50%) in the isolated diatoms, indicating a wide distribution in freshwater habitats. Among them, 19 strains belong to *N. palea*, which was present in water samples of all the 7 regions. *N. palea* has been found to be a widely distributed freshwater diatom in various lotic and lentic habitats [33]. About half of the isolated *Nitzschia* strains inhabited water with a temperature range from 25 to 30°C and pH range from 7.5 to 8.5.

More than 40% of the isolated diatom strains boasted a total lipid content of over 30%, suggesting diatoms are a good candidate for lipid production. Diatoms mainly store carbon and energy in the form of lipids, especially under stress [14]. The lipid content in 21% of the isolated *Nitzschia* and *Fistulifera* strains amounted to over 40%, which makes our screening for high oleaginous diatoms possible.

**Fig. 4:**
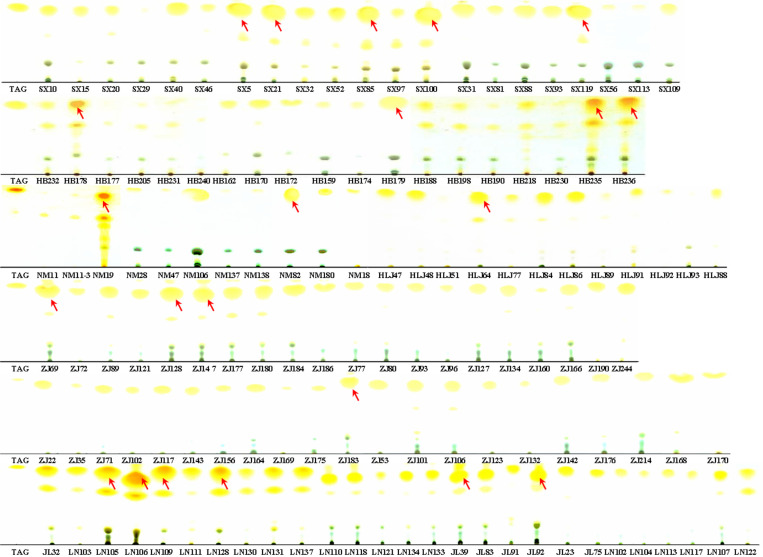
Comparison of triacylglycerol (TAG) contents in diatoms isolated from different regions in China by TLC (TAG: 0.02 mg triolein). Strains with relatively high TAG content are indicated by arrows.

TAGs, which can be converted via a transesterification process, are believed to be superior to other lipids for biodiesel production [34]. In this study, total lipids from the 131 isolated diatom strains were tested preliminarily by TLC for their TAG contents (Fig. 4). According to the images of TLC (Fig. 4), a band corresponding to standard triolein was clearly visible in all samples. Therefore, all isolated diatoms contained TAG though the contents were widely different. Based on the gray intensity of TLC images, 5 strains (*Fistulifera* sp. SX5, *Fistulifera* sp. SX100, *Nitzschia* sp. SX21, *Cyclotella* sp. SX119 and *Achnanthidium* sp. SX85) from Shanxi, 4 strains (*Fistulifera* sp. HB179, *Fistulifera* sp. HB236, *Nitzschia* sp. HB178 and *Nitzschia* sp. HB235) from Hebei, 2 strains (*Fistulifera* sp. NM19 and *Fistulifera* sp. NM82) from Inner Mongolia, one strain (*Nitzschia* sp. HLJ64) from Heilongjiang, 4 strains (*Sellaphora* sp. ZJ69, *Sellaphora* sp. ZJ147, *Achnanthidium* sp. ZJ128 and *Nitzschia* sp. ZJ183) from Zhejiang, 2 strains (*Nitzschia* sp. JL39 and *Cyclotella* sp. JL92) from Jilin, 4 strains (*Nitzschia* sp. LN109, *Nitzschia* sp. LN128, and unidentified strains LN105 and LN106) from Liaoning showed relatively higher TAG contents. Especially, strains HB235, HB 236, NM19, ZJ69 and LN106 had rather high TAG contents.

### Screening of High Temperature-Tolerant Diatoms

After preliminary experiments, a total of 49 isolated diatom strains could survive at 30°C, and they were designated as high temperature-tolerant diatoms. The 49 strains included 26 *Nitzschia*, 9 *Fistulifera*, 6 *Sellaphora*, 3 *Achnanthidium*, 1 *Cyclotella* and 3 unknown species. Twenty-nine strains out of the 49 high temperature-tolerant ones were isolated from waters at above 25°C, and among them 7 were from waters at above 30°C. Although diatoms prefer low temperatures, screening diatom strains with high temperature tolerance in hot seasons is also feasible. For example, *Nitzschia* sp. ZJ183 and *Nitzschia* sp. ZJ184 were isolated at around 30 °C, and *Cyclotella* sp. HB162, *N. palea* HB170 and *Fistulifera* sp. HB236 were from waters of 23-26°C. However, *Nitzschia* sp. HLJ48 was isolated from waters below 20°C, and it could also survive at 30°C.

**Fig. 5:**
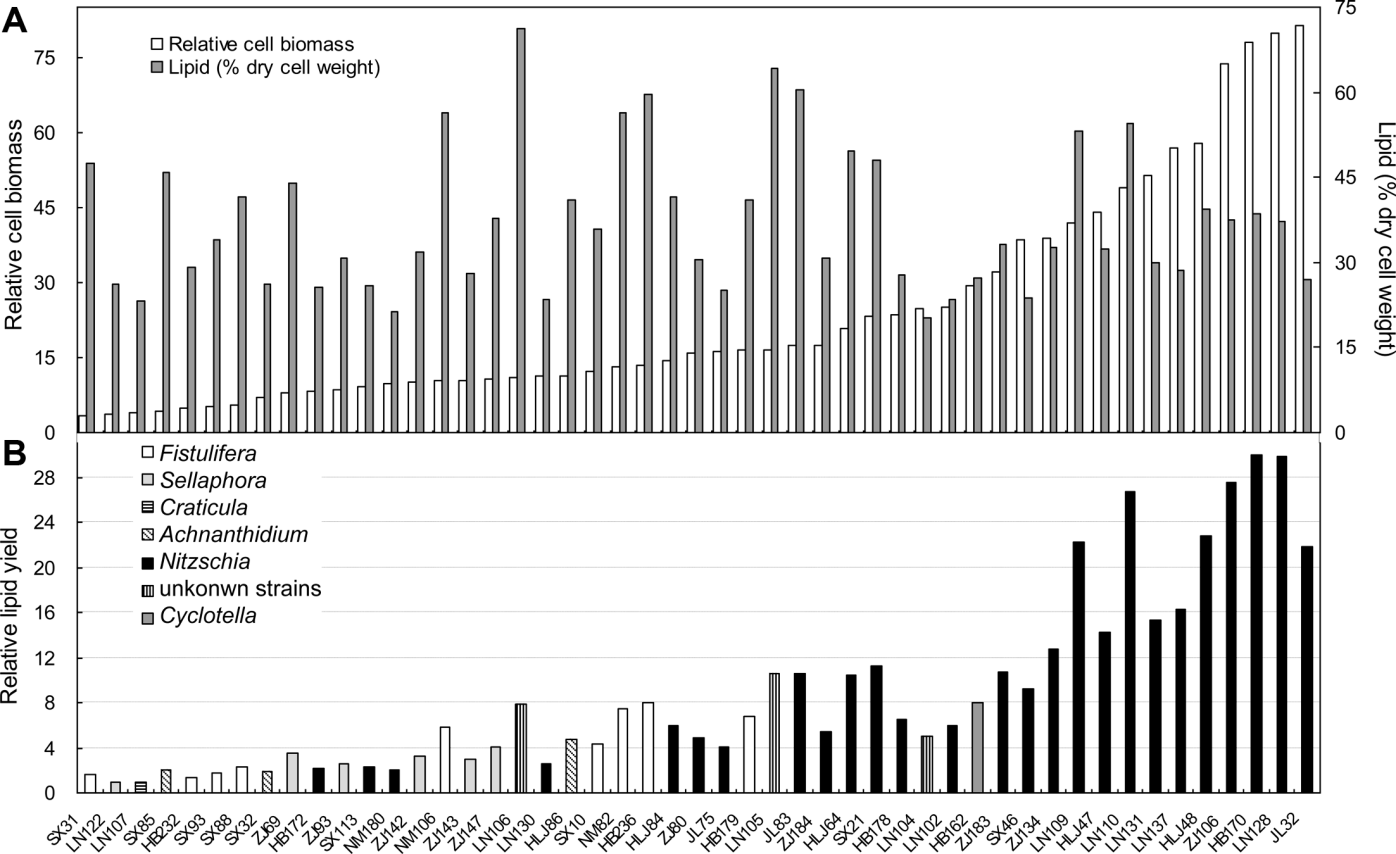
Relative cell biomass, lipid content (A) and relative lipid yield (B) of high-temperature-tolerant diatoms grown at 28°C for 10 days. Relative cell biomass was calculated based on the cell density and cell area, and relative lipid yield was calculated based on the relative cell biomass and lipid content.

Growth and lipid yields of the 49 high temperature-tolerant diatoms were compared at 28°C (Fig. 5). Strains from genus *Nitzschia* (including HB170, HLJ48 and ZJ183) showed higher relative cell biomass, while *Fistulifera* sp. HB236 and *Nitzschia* sp. JL83 had the highest total lipid content in all the 49 diatoms except for the two unidentified strains LN105 and LN106. Genera *Achnanthidium*, *Craticula* and *Sellaphora* showed much lower lipid yields compared with the others. In contrast, thirteen *Nitzschia* strains showed much higher relative lipid yields, and strain HB170 had the highest relative lipid yield. HB236 had the highest lipid yield in all 9 high temperature-tolerant *Fistulifera* strains, and HB162 was the only high temperature-tolerant strain of genus *Cyclotella*. *Nitzschia* sp. ZJ184 had a relative low lipid yield among all the high temperature-tolerant *Nitzschia* strains, and was used as a control in this study. Therefore, *Fistulifera* sp. HB236, *Cyclotella* sp. HB162 and four *Nitzschia* strains (HB170, HLJ48, ZJ183 and ZJ184) were selected for further detailed research.

### Temperature Adaptability of 6 Representative Diatom Strains

**Fig. 6:**
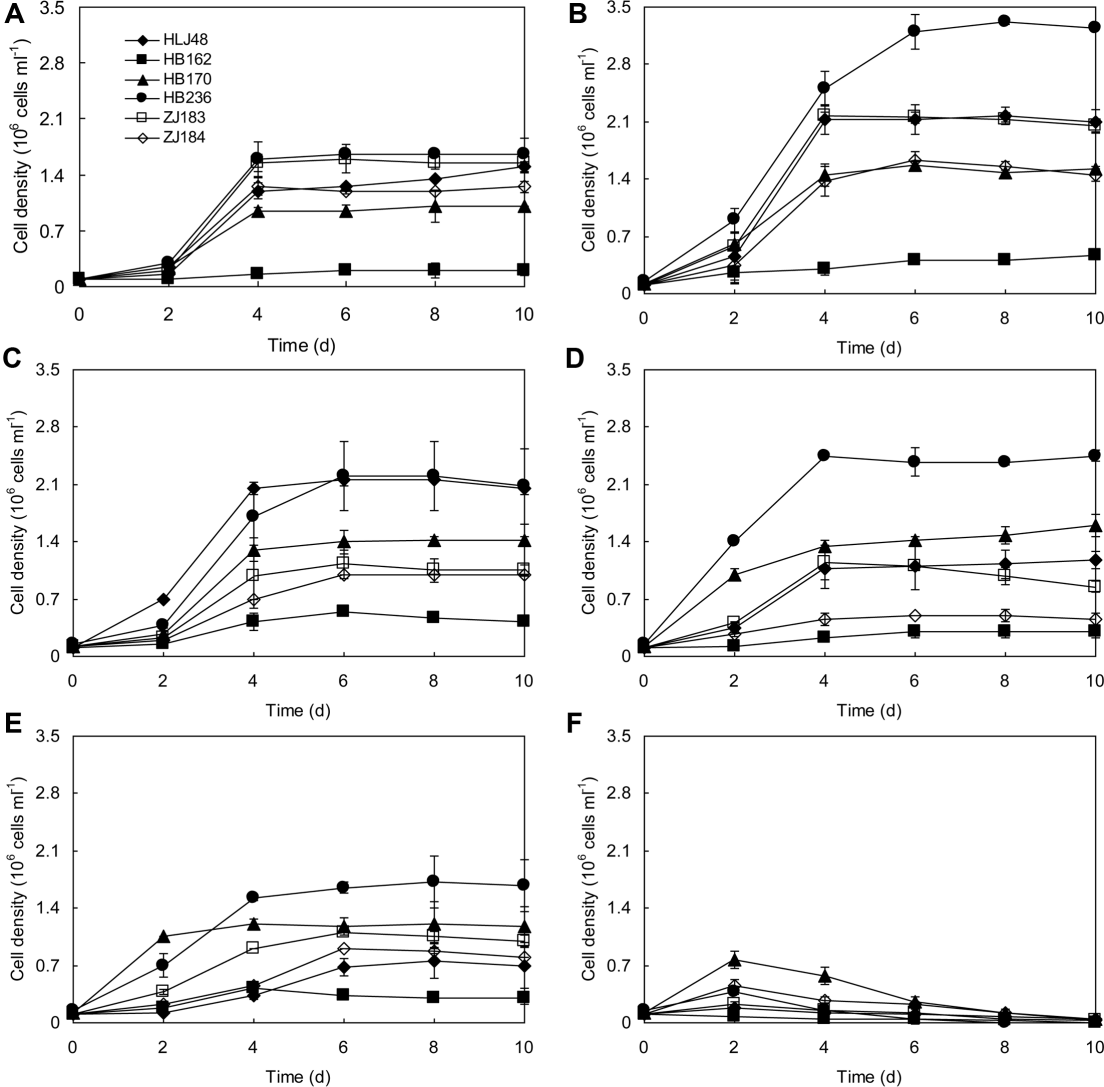
Growth curves of six representative diatoms at different temperatures (A: 22°C; B: 25°C; C: 28°C; D: 32°C; E: 35°C; F: 40°C).

**Fig. 7:**
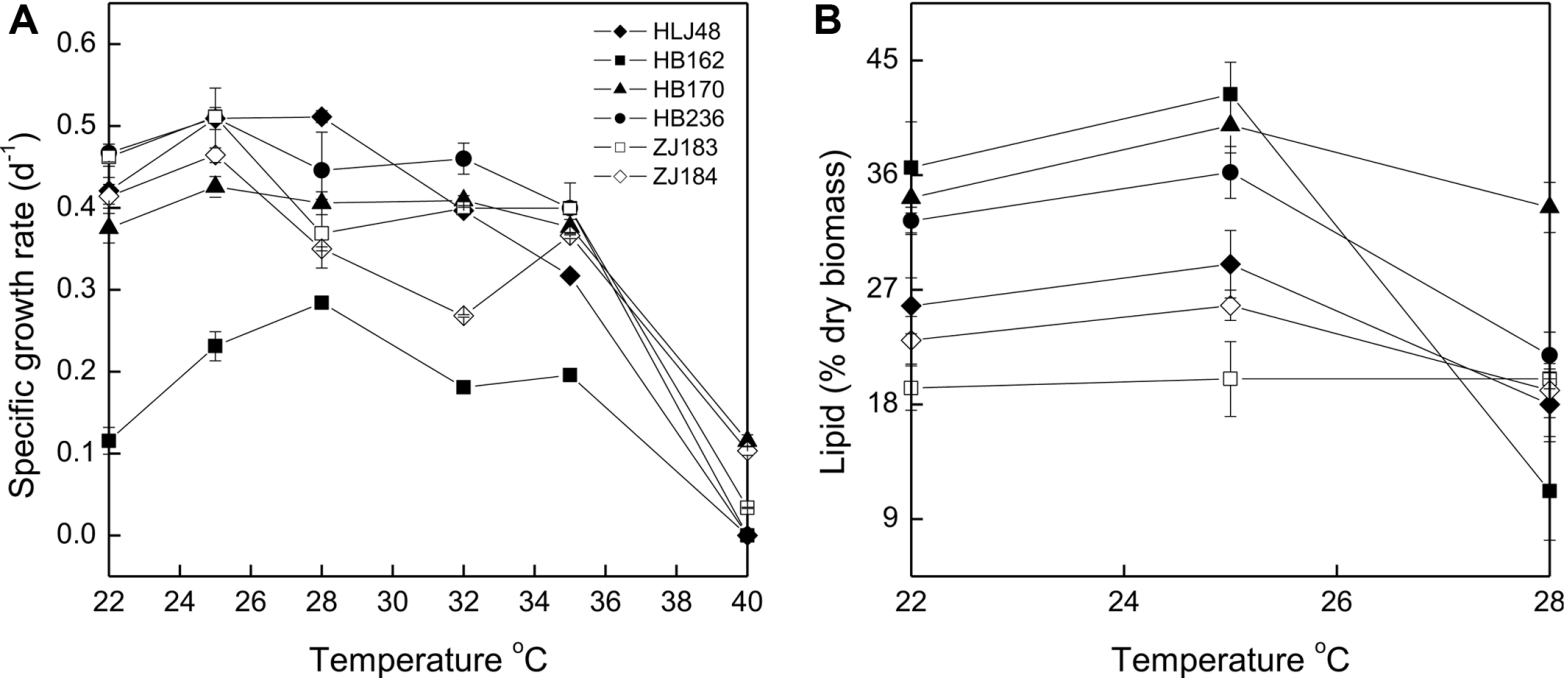
Specific growth rates (A) and total lipid contents (B) of six representative diatoms at different temperatures.

Six selected diatoms were cultivated at temperatures ranging from 22 to 40°C to observe their response to temperatures (Fig. 6). All strains could grow at temperatures from 22 to 35°C, but were incapable of normal growth at 40°C. It was reported that temperatures higher than 35°C are usually lethal for a number of species [35]. *Fistulifera* sp. HB236 and 4 *Nitzschia* strains not only grew best but also showed the highest lipid content at 25°C, however the optimum growth temperature of *Cyclotella* sp. HB162 was 28°C, though its lipid content was sharply decreased at that temperature (Fig. 7). *Cyclotella meneghiniana* has optimum growth at 25°C, and an obvious decrease occurred at 30°C [36]. The highest cell density of 42.46 × 10^4^ cells/ml was achieved in *Cyclotella* sp. FACHB-1677 with a specific growth rate of 0.188 d-1 at 30°C [37]. Although its cell density is comparable to that in *Cyclotella* sp. HB162 in our study, its specific growth rate is much lower. Jiang *et al*. [38] revealed that the optimal season for *Nitzschia* sp. to produce biomass was summer (23-37°C) but the best lipid production occurred in winter (2-17°C). *Fistulifera solaris* was well adapted to high-temperature environments, and even in summer when average daily ambient temperature exceeded 30°C and temporarily reached 42°C, steady biomass (0.31-0.57 g/l) and lipid content (18%) of the microalgae were observed under outdoor mass cultivation [39]. In this study, a dramatic decrease in cell density was observed when the 6 strains were cultivated at 35°C, but the specific growth rates of most of these diatoms at 35°C are comparable with those at 32°C. In particular, *N. palea* HB170 and *Fistulifera* sp. HB236 presented a small variation of specific growth rates within the scope of 28~35°C, thus having excellent temperature adaptability.

### Lipid Yields and Fatty Acid Compositions of 6 Representative Diatom Strains

**Fig. 8:**
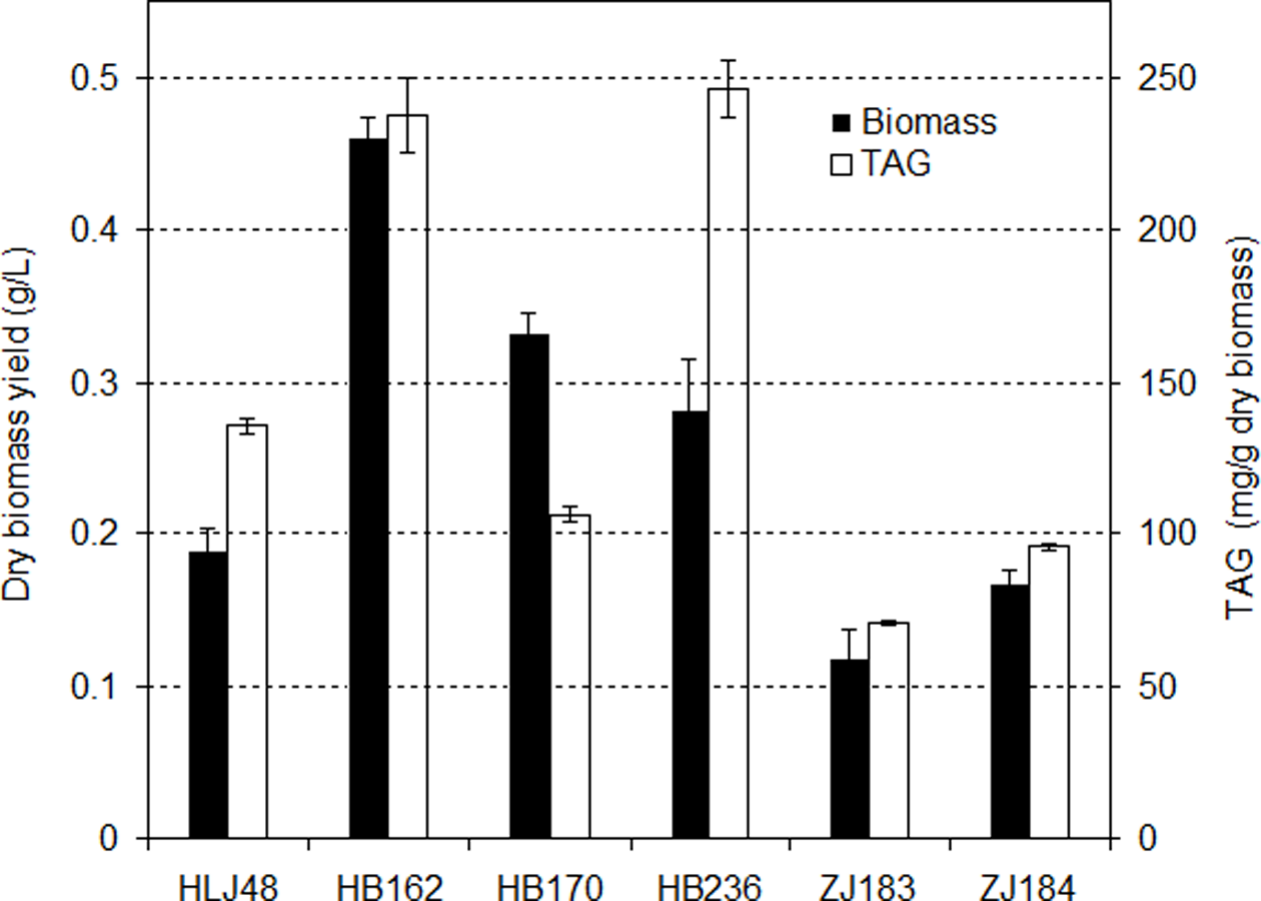
The dry biomass and TAG contents of six representative diatoms grown under optimal temperature conditions.

Fig. 8 showed the dry biomass and TAG contents of 6 diatom strains grown at 25°C for 10 days. Although its cell density was the lowest, *Cyclotella* sp. HB162 had the highest dry biomass of 0.46 g/l and relatively high TAG content of 237.4 mg/g dry biomass. The highest TAG content of 246.4 mg/g dry biomass was obtained in *Fistulifera* sp. HB236. The cell sizes (areas) of HB162 were the biggest (235 ± 18 μm^2^) among all high temperature-tolerant diatoms, and were as much as 7 times bigger than that in strain ZJ183 (28 ± 2 μm^2^). Cell sizes (areas) of strains HLJ48, HB236 and ZJ184 were 34 ± 3, 36 ± 6, and 39 ± 3 μm^2^, and that of strain HB170 was 84 ± 8 μm^2^. Thus, the dry biomass at day 10 was much lower in strains ZJ183 (0.12 g/l), ZJ184 (0.17 g/l), and HLJ48 (0.19 g/l). Since the highest cell density was observed in strain HB236 at 25°C, the relatively high dry biomass (0.28 g/l) was also obtained in this strain. *N. palea* HB170 had high dry biomass (0.33 g/l) but relatively low TAG content (105.9 mg/g dry biomass). TAG contents in strains ZJ183, ZJ184 and HLJ48 were 70.7, 95.5, and 135.9 mg/g dry biomass. Therefore, the highest TAG yield of 109.4 mg/l was obtained in strain HB162 and the lowest of 8.3 mg/l in strain ZJ183.

Besides high lipid content, an adequate fatty acid profile is indispensable in microalgae as a renewable feedstock for biodiesel production [18, 19]. The fatty acid composition of 6 diatom strains possessed a classical characteristic of class bacillariophyceae, which was mainly composed of C14:0, C16:0, C16:1, C18:0, and C20:5 (Table 1). These main fatty acids accounted for 86-97% of total fatty acids in both total lipids and TAGs. The percentages of SAFA and MUFA in the 6 strains were 84-91% in total lipids and 90-94% in TAGs, respectively. Accordingly, polyunsaturated fatty acid (PUFA) content was lower than 10% in TAGs of all 6 selected diatom strains, which makes these diatoms the ideal feedstock for biodiesel according to the requirements of the European Standard EN 14214 [40]. In particular, HB162 had rather high SAFA and MUFA percentages in both total lipids (90%) and TAGs (92%). The effects of temperature on the degree of fatty acid saturation in various diatoms have been studied extensively [16, 41-46) and lower temperature increased the contents of PUFA. Only a few studies on diatoms of genus *Amphora*, whose fatty acid profile is different from that of other diatoms, reported that the PUFAs were higher at the higher temperature [43, 47]. Although the percentage of SAFA and MUFA in our 6 strains was rather high, it is worth investigating the effects of temperature on fatty acid compositions of these strains and optimizing the content and composition of fatty acids.

In conclusion, diatoms store excess photosynthate as lipids instead of carbohydrates, and are recognized as very promising microalgae for biofuel production. However, diatoms have a preference for low temperatures and usually could not survive at high temperatures, making them unsuitable for outdoor cultivation. In this study, 131 diatom strains were isolated from China in summer, and among them, 49 strains could survive at 30°C. Six screened diatoms with relatively high biomass and lipid content out of the 49 strains could grow at 35°C, but showed different responses in growth and TAG yield to high temperatures. *N. palea* HB170 and *Fistulifera* sp. HB236 presented relatively stable growth rate and TAG yield under fluctuating temperatures ranging from 28 to 35°C, while *Cyclotella* HB162 can be used as an oil source at temperatures below 25°C. Our results demonstrate that strains HB170 and HB236 are highly desirable candidates for outdoor cultivation, while strain HB162 may be a better choice under a relatively low temperature.

## Supplemental Materials



Supplementary data for this paper are available on-line only at http://jmb.or.kr.

## Figures and Tables

**Table 1:** Fatty acid (FA) compositions (% of total fatty acids) of total lipids and triacylglycerols (TAGs) in six diatoms.

FA	HLJ48		HB162		HB170		HB236		ZJ183		ZJ184	

	Total lipids	TAGs	Total lipids	TAGs	Total lipids	TAGs	Total lipids	TAGs	Total lipids	TAGs	Total lipids	TAGs
C14:0	9.52±0.15	6.37±0.55	12.8±0	18.15±0.21	11.17±0.52	12.99±0.68	11.72±0.17	7.08±0.02	7.7±0.27	7.17±0.04	8.81±0.05	9.53±0.21
C14:1	2.21±0.38	0.44±0.32	1.94±0.11	0.38±0.2	3.34±0.56	0.18±0.09	0.65±0.05	0.66±0.06	1.26±0.07	1.04±0.05	0.45±0.15	0±0
C16:0	34.05±0.79	39.54±1.69	26.65±0.75	30.07±0.7	28.87±0.27	30.36±1.26	32.99±0.37	34.99±0.01	38.26±0.29	41.92±0.99	28.66±0.67	35.74±0.43
C16:1	34.51±0.46	34.15±0.5	34.59±0.57	41.58±0.57	37.38±0.09	44.77±0.4	35.07±0.52	41.67±0.5	34.49±0.44	33.19±0.35	35.65±0.4	41.05±0.04
C18:0	6.29±0.01	5.99±1.14	12.29±0.25	1.76±0.17	1.64±0.04	1.61±0.6	4.05±0.66	2.91±0.08	8.37±0.59	6.75±0.41	10.07±0.32	4.45±0.19
C18:1	1.2±0.12	3.65±0.17	1.35±0.68	0.16±0.07	0.76±0.03	0.99±0.15	0.45±0.01	2.6±0.48	0.55±0.03	0.67±0.07	0.86±0.26	2.47±0.02
C18:2	0.87±0.66	3.57±0.94	0.14±0.17	0.99±0.02	1.5±0.02	1.23±0.33	0.18±0.02	1.57±0.32	0.45±0.01	0.69±0.02	0.47±0.12	1.48±0.04
C18:3	0.65±0.2	0.34±0.19	0.49±0.04	0.83±0.15	3.08±0.33	1.83±0.08	0.77±0.03	0.63±0.12	0.57±0.01	1.03±0.19	1.41±0.5	3.25±0.01
C18:4	0.19±0	0.31±0.01	0.78±0.08	0.82±0.05	0.32±0.45	0.93±0	0.2±0.01	0.4±0.03	0.32±0	0.34±0.07	0.9±0.06	0±0
C20:0	0.06±0	0.25±0.04	0.1±0	0.32±0.05	0.66±0.94	0.02±0.02	0.11±0	0.3±0.17	0±0	0.19±0.01	0±0	0.8±0.11
C20:4	0.94±0.02	0.75±0	0.04±0.03	0±0	1.55±0.07	0.98±0	1.11±0.03	0.72±0.05	0.8±0.03	1.22±0.11	0.84±0.08	0.23±0.2
C20:5	9.19±0.55	4.61±0.74	8.64±0.07	4.93±0.65	7.33±0.17	4.11±0.08	12.7±0.02	6.39±0.21	7.23±0.19	5.8±0.01	11.86±0.19	0.89±0.01
C22:5	0.31±0.08	0.04±0.05	0.19±0.03	0±0	2.39±0.01	0±0	0±0	0.07±0.10	0±0	0±0	0±0	0.09±0.07
SAFA	49.92	52.15	51.84	50.3	42.34	44.98	48.87	45.28	54.33	56.03	47.54	50.52
MUFA	37.92	38.24	37.88	42.12	41.48	45.94	36.17	44.93	36.3	34.9	36.96	43.52
PUFA	12.15	9.62	10.28	7.57	16.17	9.08	14.96	9.78	9.37	9.08	15.48	5.94

SAFA: saturated FA; MUFA: monounsaturated FA; PUFA: polyunsaturated FA.
