# Supplementary material for: Screening of High Temperature-Tolerant Oleaginous Diatoms
Source: J Microbiol Biotechnol. 2020 Apr 23;30(7):1072–81. doi: 10.4014/jmb.2002.02053 (PMC9728242; doi:10.4014/jmb.2002.02053)
Supplement: Supplementary file 1 [file JMB-30-7-1072-supple.pdf]

J. Microbiol. Biotechnol.
https://doi.org/10.4014/jmb.2002.02053

Name	Genus	Habitats
SX5	*Fistulifera* sp.	pH(7.85)Cond(2610 μs/cm)T(27.5)
SX10	*Fistulifera* sp.	pH(9.72)Cond(331 μs/cm)T(29.5)
SX15	*Nitzschia* sp.	pH(8.8)Cond(627 μs/cm)T(25.9)
SX20	*Nitzschia* sp.	pH(8.85)Cond(841 μs/cm)T(27.5)
SX21	*Nitzschia* sp.	pH(8.41)Cond(849 μs/cm)T(24.6)
SX29	*Fistulifera* sp.	pH(7.85)Cond(851 μs/cm)T(24.8)
SX31	*Fistulifera* sp.	pH(8.56)Cond(465 μs/cm)T(28.5)
SX32	*Achnanthidium* sp.	pH(8.46)Cond(471 μs/cm)T(26.9)
SX40	*Cyclotella* sp.	pH(8.54)Cond(521 μs/cm)T(28.5)
SX46	*Nitzschia* sp.	pH(8.97)Cond(452 μs/cm)T(28.2)
SX52	*Synedra* sp.	pH(8.20)Cond(480 μs/cm)T(22.6)
SX56	*Nitzschia* sp.	pH(9.65)Cond(327 μs/cm)T(25.2)
SX81	*Fistulifera* sp.	pH(8.24)Cond(587 μs/cm)T(26.3)
SX85	*Achnanthidium* sp.	pH(8.93)Cond(658 μs/cm)T(27.2)
SX88	*Fistulifera* sp.	pH(9.32)Cond(1542 μs/cm)T(26.1)
SX93	*Fistulifera* sp.	pH(8.47)Cond(477 μs/cm)T(18.2)
SX97	*Fistulifera* sp.	pH(8.35)Cond(580 μs/cm)T(23.4)
SX100	*Fistulifera* sp.	pH(8.57)Cond(407 μs/cm)T(22.9)
SX109	*Achnanthidium* sp.	pH(8.86)Cond(1267 μs/cm)T(29.8)
SX113	*Nitzschia* sp.	pH(8.91)Cond(1024 μs/cm)T(33.2)
SX119	*Cyclotella* sp.	pH(9.13)Cond(3440 μs/cm)T(28.9)
HB159	*Fistulifera* sp.	pH(9.11)Cond(621 μs/cm)T(24.2)
HB162	*Cyclotella* sp.	pH(9.62)Cond(1177 μs/cm)T(24.6)
HB170	*Nitzschia* sp.	pH(8.71)Cond(1248 μs/cm)T(25.5)
HB172	*Nitzschia* sp.	pH(8.87)Cond(952 μs/cm)T(26.6)
HB174	*Fistulifera* sp.	pH(9.02)Cond(942 μs/cm)T(23.8)
HB177	*Fistulifera* sp.	pH(8.87)Cond(423 μs/cm)T(19.7)
HB178	*Nitzschia* sp.	pH(8.43)Cond(549 μs/cm)T(12.8)
HB179	*Fistulifera* sp.	pH(7.6)Cond(578 μs/cm)T(10.5)
HB188	*Nitzschia* sp.	pH(7.83)Cond(1036 μs/cm)T(28.7)
HB190	*Fistulifera* sp.	pH(7.99)Cond(502 μs/cm)T(25.2)
HB198	*Sellaphora* sp.	pH(7.68)Cond(906 μs/cm)T(21.5)
HB205	*Fistulifera* sp.	pH(8.43)Cond(412 μs/cm)T(25.3)
HB218	*Fistulifera* sp.	pH(7.96)Cond(1602 μs/cm)T(25.9)
HB230	*Nitzschia* sp.	pH(8.88)Cond(573 μs/cm)T(26.6)
HB231	*Fistulifera* sp.	pH(9.33)Cond(560 μs/cm)T(29.2)
HB232	*Nitzschia* sp.	pH(9.09)Cond(561 μs/cm)T(28.6)
HB235	*Nitzschia* sp.	pH(8.14)Cond(736 μs/cm)T(25.5)
HB236	*Fistulifera* sp.	pH(8.09)Cond(771 μs/cm)T(22.9)
HB240	*Nitzschia* sp.	pH(8.17)Cond(595 μs/cm)T(25.1)
NM11	*Nitzschia* sp.	nd.
NM11-3	*Nitzschia* sp.	nd.
NM19	*Fistulifera* sp.	nd.
NM28	*Nitzschia* sp.	pH(9.9)Cond(6460 μs/cm)T(25.2)

NM47	*Nitzschia* sp.	pH(7.56)Cond(376 μs/cm)T(25)
NM82	*Fistulifera* sp.	pH(8.11)Cond(888 μs/cm)T(17.4)
NM106	*Fistulifera* sp.	nd.
NM137	*Nitzschia* sp.	nd.
NM138	*Fistulifera* sp.	nd.
NM180	*Fistulifera* sp.	nd.
NM18	*Nitzschia* sp.	pH(7.91)Cond(164 μs/cm)T(19.5)
HLJ47	*Nitzschia* sp.	pH(7.37)Cond(518 μs/cm)T(22.6)
HLJ48	*Nitzschia* sp.	pH(7.16)Cond(607 μs/cm)T(19.5)
HLJ51	*Nitzschia* sp.	pH(7.62)Cond(367 μs/cm)T(23.4)
HLJ64	*Nitzschia* sp.	pH(7.48)Cond(545 μs/cm)T(25)
HLJ77	*Nitzschia* sp.	pH(7.98)Cond(69400 μs/cm)T(25.7)
HLJ84	*Nitzschia* sp.	pH(7.6)Cond(700 μs/cm)T(22.9)
HLJ86	*Achnanthidium* sp.	pH(7.95)Cond(13950 μs/cm)T(23.9)
HLJ88	*Nitzschia* sp.	pH(8.87)Cond(87000 μs/cm)T(25.6)
HLJ89	*Nitzschia* sp.	pH(7.93)Cond(101100 μs/cm)T(26.7)
HLJ91	*Nitzschia* sp.	pH(8.5)Cond(66100μs/cm)T(25.4)
HLJ92	*Nitzschia* sp.	pH(7.65)Cond(11650μs/cm)T(21.8)
HLJ93	*Nitzschia* sp.	pH(7.65)Cond(11650μs/cm)T(21.8)
ZJ22	*Achnanthidium* sp.	pH(7.47)Cond(46700μs/cm)T(26.4)
ZJ35	*Sellaphora* sp.	pH(8.14)Cond(17020μs/cm)T(28.3)
ZJ53	*Pinnularia* sp.	pH(7.6)Cond(86.3μs/cm)T(26.6)
ZJ69	*Sellaphora* sp.	pH(7.57)Cond(62.1μs/cm)T(25.8)
ZJ71	*Pinnularia* sp.	pH(8.7)Cond(70.3μs/cm)T(29.2)
ZJ72	*Nitzschia* sp.	pH(7.5)Cond(42.4μs/cm)T(28.2)
ZJ77	*Pinnularia* sp.	pH(7.8)Cond(63.7μs/cm)T(27.9)
ZJ80	*Nitzschia* sp.	pH(7.46)Cond(134.1μs/cm)T(24.4)
ZJ89	*Nitzschia* sp.	pH(8.5)Cond(27.4μs/cm)T(27.9)
ZJ93	*Sellaphora* sp.	pH(7.89)Cond(9.81μs/cm)T(23.7)
ZJ96	*Nitzschia* sp.	pH(7.78)Cond(7.49μs/cm)T(34.2)
ZJ101	unknown	pH(8.1)Cond(7.01μs/cm)T(27.1)
ZJ102	*Nitzschia* sp.	pH(7.61)Cond(9.93μs/cm)T(26.9)
ZJ106	*Nitzschia* sp.	pH(10.26)Cond(34.6μs/cm)T(28.7)
ZJ117	*Achnanthidium* sp.	pH(9.62)Cond(24.6μs/cm)T(31)
ZJ121	*Lemnicola* sp.	pH(7.6)Cond(21.7μs/cm)T(33.2)
ZJ123	*Achnanthidium* sp.	pH(8.2)Cond(22.7μs/cm)T(33.8)
ZJ127	*Achnanthidium* sp.	pH(8.99)Cond(54.1μs/cm)T(33.3)
ZJ128	*Achnanthidium* sp.	pH(9.1)Cond(37.7μs/cm)T(33.2)
ZJ132	*Achnanthidium* sp.	pH(8.25)Cond(27.3μs/cm)T(28.4)
ZJ134	*Nitzschia* sp.	pH(7.95)Cond(9.27μs/cm)T(23.4)
ZJ142	*Sellaphora* sp.	pH(8.57)Cond(32.3μs/cm)T(29.1)
ZJ143	*Sellaphora* sp.	pH(8.17)Cond(28.8μs/cm)T(30.9)
ZJ147	*Sellaphora* sp.	pH(9.51)Cond(45.9μs/cm)T(31.9)
ZJ156	*Nitzschia* sp.	pH(7.83)Cond(58.1μs/cm)T(30.4)
ZJ160	*Sellaphora* sp.	pH(9.03)Cond(43.6μs/cm)T(33.4)

ZJ164	unknown	pH(8.8)Cond(29.6μs/cm)T(31.4)
ZJ166	*Achnanthidium* sp.	pH(8.33)Cond(27.4μs/cm)T(32.5)
ZJ168	*Nitzschia* sp.	pH(10.39)Cond(50.4μs/cm)T(33.8)
ZJ169	*Gomphonema* sp.	pH(8.89)Cond(46.6μs/cm)T(31.7)
ZJ170	*Nitzschia* sp.	pH(8.36)Cond(59.6μs/cm)T(31.9)
ZJ175	*Achnanthidium* sp.	pH(9.27)Cond(47.5μs/cm)T(31.5)
ZJ176	*Nitzschia* sp.	pH(7.43)Cond(63.1μs/cm)T(31.8)
ZJ177	*Lemnicola* sp.	pH(7.37)Cond(74.6μs/cm)T(28.8)
ZJ180	*Nitzschia* sp.	pH(7.77)Cond(74.7μs/cm)T(31.2)
ZJ183	*Nitzschia* sp.	pH(8.06)Cond(69.1μs/cm)T(29.9)
ZJ184	*Nitzschia* sp.	pH(9.18)Cond(28.8μs/cm)T(30.1)
ZJ186	*Achnanthidium* sp.	pH(7.8)Cond(30.5μs/cm)T(30)
ZJ190	*Nitzschia* sp.	pH(6.65)Cond(37.2μs/cm)T(30.4)
ZJ214	*Nitzschia* sp.	pH(6.49)Cond(116.6μs/cm)T(27.5)
ZJ244	*Nitzschia* sp.	pH(8.59)Cond(56μs/cm)T(30.3)
JL23	*Cyclotella* sp.	pH(6.60)Cond(153.9μs/cm)T(24.6)
JL32	*Nitzschia* sp.	pH(7.20)Cond(131.2μs/cm)T(25.5)
JL39	*Nitzschia* sp.	pH(7.10)Cond(83.0μs/cm)T(26.5)
JL75	*Nitzschia* sp.	pH(7.55)Cond(70.1μs/cm)T(28.0)
JL83	*Nitzschia* sp.	pH(7.00)Cond(53.3μs/cm)T(27.6)
JL91	*Cyclotella* sp.	pH(7.50)Cond(54.2μs/cm)T(26.2)
JL92	*Cyclotella* sp.	pH(8.07)Cond(41.6μs/cm)T(25.7)
LN102	*Nitzschia* sp.	pH(6.72)Cond(64.3μs/cm)T(23.1)
LN103	*Nitzschia* sp.	pH(7.22)Cond(28.1μs/cm)T(19.4)
LN104	unknown	pH(6.98)Cond(25.0μs/cm)T(19.8)
LN107	*Craticula* sp.	pH(8.17)Cond(54.0μs/cm)T(26.5)
LN109	*Nitzschia* sp.	pH(8.40)Cond(68.2μs/cm)T(28.7)
LN110	*Nitzschia* sp.	pH(6.52)Cond(47.8μs/cm)T(23.4)
LN111	*Cyclotella* sp.	pH(6.72)Cond(93.6μs/cm)T(26.2)
LN113	*Nitzschia* sp.	pH(7.74)Cond(81.5μs/cm)T(28.6)
LN117	*Nitzschia* sp.	pH(7.40)Cond(90.1μs/cm)T(29.6)
LN118	*Nitzschia* sp.	pH(7.61)Cond(74.9μs/cm)T(30.3)
LN121	*Nitzschia* sp.	pH(7.77)Cond(64.6μs/cm)T(29.8)
LN122	*Sellaphora* sp.	pH(7.61)Cond(62.4μs/cm)T(29.2)
LN128	*Nitzschia* sp.	pH(7.57)Cond(83.6μs/cm)T(26.1)
LN130	*Nitzschia* sp.	pH(7.74)Cond(62.5μs/cm)T(30.3)
LN131	*Nitzschia* sp.	pH(7.96)Cond(66.0μs/cm)T(30.4)
LN133	*Sellaphora* sp.	pH(7.67)Cond(112.7μs/cm)T(30.9)
LN134	*Nitzschia* sp.	pH(6.87)Cond(109.5μs/cm)T(29.3)
LN137	*Nitzschia* sp.	pH(7.87)Cond(86.4μs/cm)T(31.5)
LN105	unknown	pH(7.67)Cond(52.8μs/cm)T(27.7)
LN106	unknown	pH(7.76)Cond(53μs/cm)T(26.9)
